# A proposed method to measure weak magnetic field based on a hybrid optomechanical system

**DOI:** 10.1038/s41598-017-12639-2

**Published:** 2017-10-02

**Authors:** Zeng-Xing Liu, Bao Wang, Cui Kong, Liu-Gang Si, Hao Xiong, Ying Wu

**Affiliations:** School of Physics, Huazhong University of Science and Technology, Wuhan, 430074 People’s Republic of China

## Abstract

Optomechanical systems have long been considered in the field of precision measurement. In this work, measurement of weak magnetic field in a hybrid optomechanical system is discussed. In contrast to conventional measurements based on detecting the change of magnetic flux, our scheme presents an alternative way to measure the magnetic field with a precision of 0.1 *nT*. We show that the effective cavity resonance frequency will be revised due to the electromagnetic interactions. Therefore, a resonance valley in the transmission spectrum of the probe field will shift in the presence of the magnetic field, and the width of an asymmetric transparency in the optomechanically induced transparency (OMIT) shows a strong dependence on the magnetic field strength. Our results may have potential application for achieving high precision measurement of the magnetic field.

## Introduction

Cavity optomechanics^1^, combining mechanical modes with optical modes via radiation pressure, has become a rapidly developing field recently and plays an important role in many fields of physics^2,3^, including cooling of mechanical oscillators to their motional quantum ground-state^4,5^, strong coupling physics^6^, gravitational-wave detectors^7^, manipulation of light propagation^8,9^ and radiation-mechanics entanglement^10,11^. It has been shown that many effects observed in atomic systems, like electromagnetically induced transparency^12,13^, can also occur in an optomechanical system through mechanical effects of light. A typical example is optomechanically induced transparency (OMIT) which has been demonstrated theoretically^14–16^ and experimentally^17^ in an optomechanical system. The process of OMIT is that the presence of a strong control field and a weak probe field induce an anti-Stokes field produced from the scattering of light from the control field. Destructive interference between the anti-Stokes field and the probe field can suppress the buildup of an intracavity probe field and then lead to a transparent window in transmission spectrum of the probe field. This physical process can also be intuitively explained by a Λ configuration picture constructed by the optical and mechanical modes. This interesting phenomenon provides a special platform for performing precision measurement^18–20^, such as optical mass sensor^21–23^ and precision measurement of electrical charges^24,25^.

Precision measurement is one of the most significant tasks in modern physics. The mechanical resonators hold the promise for realizing precision measurement and can be achieved by two types of systems, that is, optomechanical and electromechanical systems. Generally speaking, optomechanical measurements is considered to have higher sensitivity than electromechanical measurements^26^. In an optomechanical system, the precision measurement were usually carried out via the transmission spectrum of the probe field in OMIT. Some previous studies^25^ have shown that resonantly enhanced feedback-backaction arising from radiation pressure can be substantively modified by the Coulomb interactions, and the transparency window width reveals a strong dependence on the charge number, which makes measurement of the charge number possible. Additionally, a tunable double-OMIT have been observed from the output field in an optomechanical system induced by the Coulomb interactions, which might be applied to measure the Coulomb coupling strength^27^. However, the precision measurement of a magnetic field in an optomechanical system is still unknown.

In this paper, we focus on a hybrid optomechanical system, in which one end of a moveable mirror was passed current and the whole system was placed in a magnetic field. We find that the effective cavity resonance frequency can be modified in the presence of the magnetic field. A resonance valley in the transmission curve of the probe field therefore will shift due to the electromagnetic interactions. Furthermore, the steady-state position of the mechanical oscillator (MO) will be affected coram the electromagnetic interactions and the transparency window of the OMIT will change correspondingly. Our scheme shows that an asymmetric transparency window appears in the transmission spectrum of the probe field and also the width of this window can be tunable by the electromagnetic interactions. Additionally, the current intensity plays a crucial role in our study, which determines the relationship between the probe power transmission and the magnetic field strength. As a result, we believe that our scheme enables a potentially practical proposal for precision measurement of the magnetic field.

## Results

### Model, Hamiltonian, steady states and the probe power transmission

**Figure 1:**
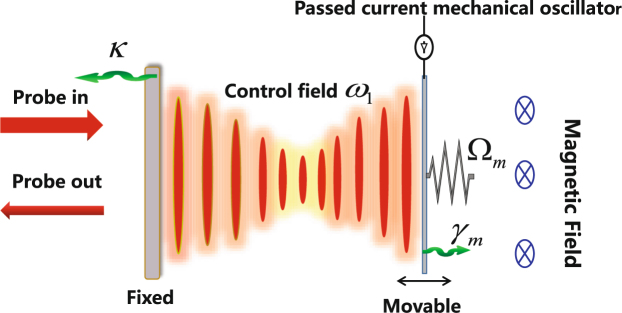
Schematic of the system. A hybrid optomechanical system is driven by a strong coupling field with frequency *ω*_l_ and a weak probe field with frequency *ω*_p_. *κ* and *γ*_m_ are denote the decay rate of the cavity and MO, respectively. The electricity with current intensity *I* is passed into one end of our system and the whole system is placed in a magnetic field.

The physical pattern under investigation is a hybrid optomechanical system. The system consists of an optical cavity with a movable boundary, illustrated here as a high-Q Fabry-Pérot cavity in which one mirror was passed surface current, acting like a MO. The whole system was placed in a magnetic field, as sketched shown in Fig. 1. The single-mode cavity field with eigenfrequency *ω*_c_ couples to the MO with effective mass *m* and frequency Ω_m_ via radiation pressure. Now we presume that a strong driving field with frequency *ω*_l_ and a weak probe field with frequency *ω*_p_ are applied on the cavity. The Hamiltonian of the driven optomechanical system can therefore be written as^24^:

1 $$\begin{array}{rcl}\hat{H} & = & (\frac{{\hat{p}}^{2}}{2m}+\frac{1}{2}m{{\rm{\Omega }}}_{{\rm{m}}}^{2}{\hat{x}}^{2})+\hslash {\omega }_{{\rm{c}}}{\hat{a}}^{\dagger }\hat{a}+\hslash G\hat{x}{\hat{a}}^{\dagger }\hat{a}+\xi B\hat{x}\\  &  & +i\hslash \sqrt{{\eta }_{{\rm{c}}}\kappa }({\varepsilon }_{{\rm{l}}}{e}^{-i{\omega }_{{\rm{l}}}t}{\hat{a}}^{\dagger }-{\varepsilon }_{{\rm{l}}}^{\ast }{e}^{i{\omega }_{{\rm{l}}}t}\hat{a})+i\hslash \sqrt{{\eta }_{{\rm{c}}}\kappa }({\varepsilon }_{p}{e}^{-i{\omega }_{{\rm{p}}}t}{\hat{a}}^{\dagger }-{\varepsilon }_{{\rm{p}}}^{\ast }{e}^{i{\omega }_{{\rm{p}}}t}\hat{a}),\end{array}$$

where the first term describes the energy for the MO with $\hat{x}$ and $\hat{p}$ being the position and momentum operators, respectively. The second term is the free Hamiltonian for the cavity with the annihilation (creation) operator $\hat{a}$ (${\hat{a}}^{\dagger }$). The third term describes the interaction Hamiltonian between the cavity and MO with a coupling strength G. The next term presents the electromagnetic interaction Hamiltonian where *ξ* is the Amperes coefficient which is determined by the current intensity and the effective range of action. The last two terms represent the Hamiltonian of the driving fields. The coupling parameter *η*_c_ is chosen to be the critical coupling value 1/2 and *κ* the total decay rate of the cavity. ${\varepsilon }_{{\rm{i}}}=\sqrt{{P}_{{\rm{i}}}/\hslash {\omega }_{{\rm{i}}}}$ (with i = l, p) are the amplitudes of the input fields with *P*_l_ the pump power of the driving field and *P*_p_ the power of the probe field. The Heisenberg-Langevin equations can be obtain as follows:

2 $$\begin{array}{l}\frac{da}{dt}=(i{{\rm{\Delta }}}_{c}-iGx-\kappa \mathrm{/2)}a+\sqrt{{\eta }_{{\rm{c}}}\kappa }({\varepsilon }_{{\rm{l}}}+{\varepsilon }_{{\rm{p}}}{e}^{-i\delta t}),\\ \frac{dx}{dt}=\frac{p}{m},\\ \frac{dp}{dt}=-m{{\rm{\Omega }}}_{{\rm{m}}}^{2}x-\hslash G{a}^{\dagger }a+\xi B-{\gamma }_{{\rm{m}}}p,\end{array}$$

where Δ_c_ = *ω*_l_ − *ω*_c_ and *δ* = *ω*_p_ − *ω*_l_. The decay rates for the optical *κ* and MO *γ*_m_ have been introduced classically. Furthermore, the mean-field approximation is used and the quantum noise terms are dropped safely in the semiclassical approximation^1^. We first denote $\bar{a}$ and $\bar{x}$ the intracavity field and mechanical displacement for the static solution for the case that the control field is much stronger than the probe field and all time derivatives vanish. From Eq. (2), it follow immediately that $\bar{a}$ and $\bar{x}$ must fulfill the self consistent equations:

3 $$\bar{a}=\frac{\sqrt{{\eta }_{c}\kappa }{\varepsilon }_{l}}{\kappa \mathrm{/2}-i\bar{{\rm{\Delta }}}},\,\bar{x}=-\frac{\hslash G{|\bar{a}|}^{2}-\xi B}{m{{\rm{\Omega }}}_{m}^{2}},$$

where $\bar{{\rm{\Delta }}}={{\rm{\Delta }}}_{{\rm{c}}}-G\bar{x}$ is the effective cavity detuning, depending on the steady-state position $\bar{x}$ of the MO. In other words, the effective cavity frequency ${\omega }_{{\rm{c}}}-G\bar{x}$ can be adjusted by controlling the electromagnetic interaction *ξB*. It reminds us the possibility to achieve the precision measurement of the magnetic field in our scheme.

In the case of a weak probe field (compared to the driving field), the general expression of the probe power transmission, this is, the ratio of the probe power returned from the system divided by the input probe power

4 $${|{t}_{{\rm{p}}}|}^{2}={|1-\frac{1+if(\delta ,B)}{\kappa \mathrm{/2}-i(\bar{{\rm{\Delta }}}+\delta )+2\bar{{\rm{\Delta }}}f(\delta ,B)}{\eta }_{c}\kappa |}^{2},$$

where $f(\delta ,B)=\hslash {G}^{2}{|\bar{a}|}^{2}\chi (\delta )/[\kappa \mathrm{/2}+i(\bar{{\rm{\Delta }}}-\delta )]$, with $\chi (\delta )=\mathrm{1/}m({{\rm{\Omega }}}_{m}^{2}-{\delta }^{2}-i{\gamma }_{m}\delta )$ is the susceptibility of the MO. The detailed solution are described in the Methods.

In what follows, we numerically research the transmission spectrum of the probe field in our scheme. We show that the transmission curve appears a strong dependence on the electromagnetic interactions. Moreover, the measurement sensitivity can be improved by adjusting the current intensity. We believe that our study will be useful for measuring the magnetic field accurately. The parameters used in this work are analogous to those of ref.^28^ for the MO. This is *m* = 100 *pg*, *G*/2*π* = −11 MHz/nm, Ω_m_/2*π* = 0.1 MHz, *γ*_m_/2*π* = 0.2 kHz, *κ* = 0.2Ω_m_ and the wavelength of the driving field *λ*_c_ = 2*πc*/*ω*_c_ = 532 nm. We assume that the units of Amperes coefficient *ξ* = 2.0 × 10^−5^ *A* · *m* for the current intensity *I* = 1 *mA*.

### Electromagnetic interactions induced a shift of the resonance valley in the transmission spectrum of the probe field

**Figure 2:**
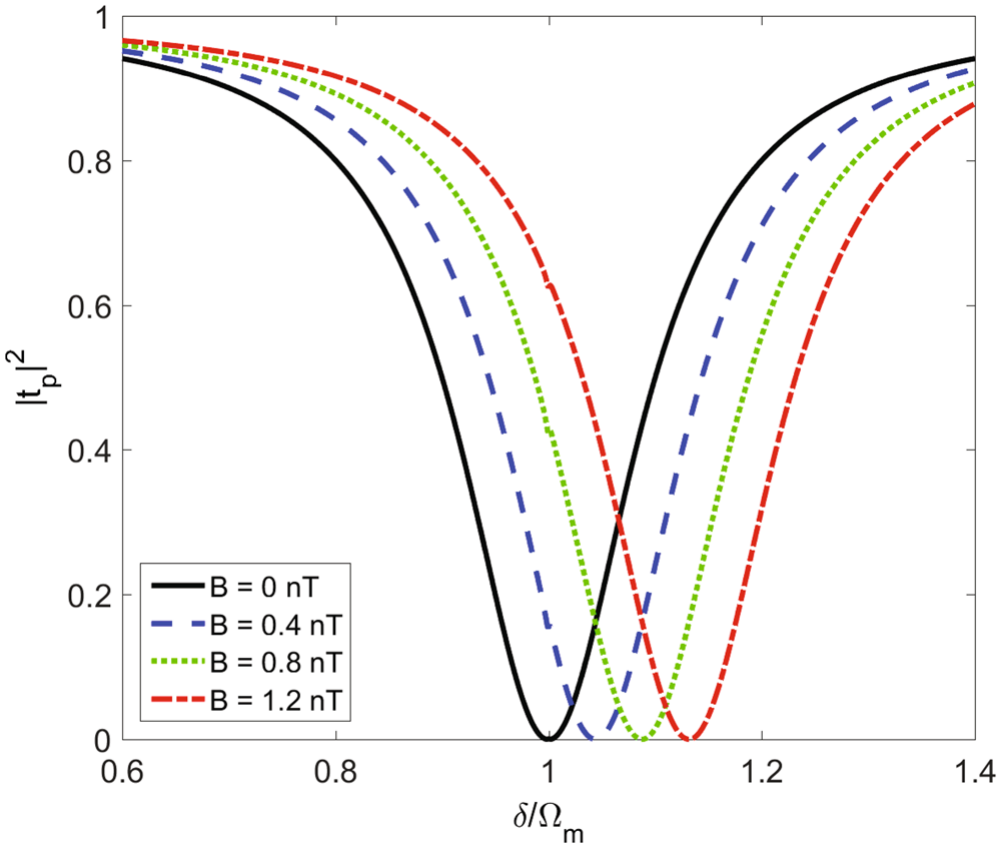
Electromagnetic interactions induced the shift of the resonance valley in the transmission spectrum of the probe field. Plots of the probe power transmission |*t*_p_|^2^ vary with *δ* in the absence (black solid line) and presence (blue dashed, green dotted, and red dash-dotted lines) of the different magnetic field strength B. we use *P*_l_ = 1 *pW*, *m* = 100 *pg*, *G*/2*π* = −11 *MHz*/*nm*, Ω_m_/2*π* = 0.1 *MHz*, *γ*_m_/2*π* = 0.2 *kHz*, *κ* = 0.2Ω _m_.

Firstly, we investigate the shift of the resonance valley in the transmission curve of the weak probe field which results from the electromagnetic interactions. In Fig. 2, we plot the probe power transmission |*t*_p_|^2^ vary with *δ* under different magnetic field strength B. It is shown that |*t*_p_|^2^ has the reversed Lorentzian-like transmission shape and the probe field is almost completely absorbed near the resonance condition. In the absence of the magnetic field *B* = 0 *nT*, i.e., without the electromagnetic interactions, the resonance valley appears at the position *δ*/Ω_m_ = 1. The resonance condition will change in the present of the electromagnetic interactions which leads to the shift of the resonance valley. As shown in Fig. 2, as the magnetic field strength *B* increases, the shift of the resonance valley becomes larger. The ultimate physical mechanism for this interesting phenomenon is as follow: The effective cavity resonance frequency ${\omega }_{{\rm{c}}}^{^{\prime} }={\omega }_{{\rm{c}}}-G\bar{x}$ shows a strong dependence on the steady-state position $\bar{x}$ which changes when we adjust the magnetic field intensity *B*. The transmission spectrum of the probe field therefore shift by the term $G\bar{x}/{{\rm{\Omega }}}_{{\rm{m}}}$ due to the electromagnetic interactions. Such electromagnetic interactions induced shift of the resonance valley might be applied to measure the magnetic field.

### Asymmetry transparency window in OMIT induced by electromagnetic interactions

**Figure 3:**
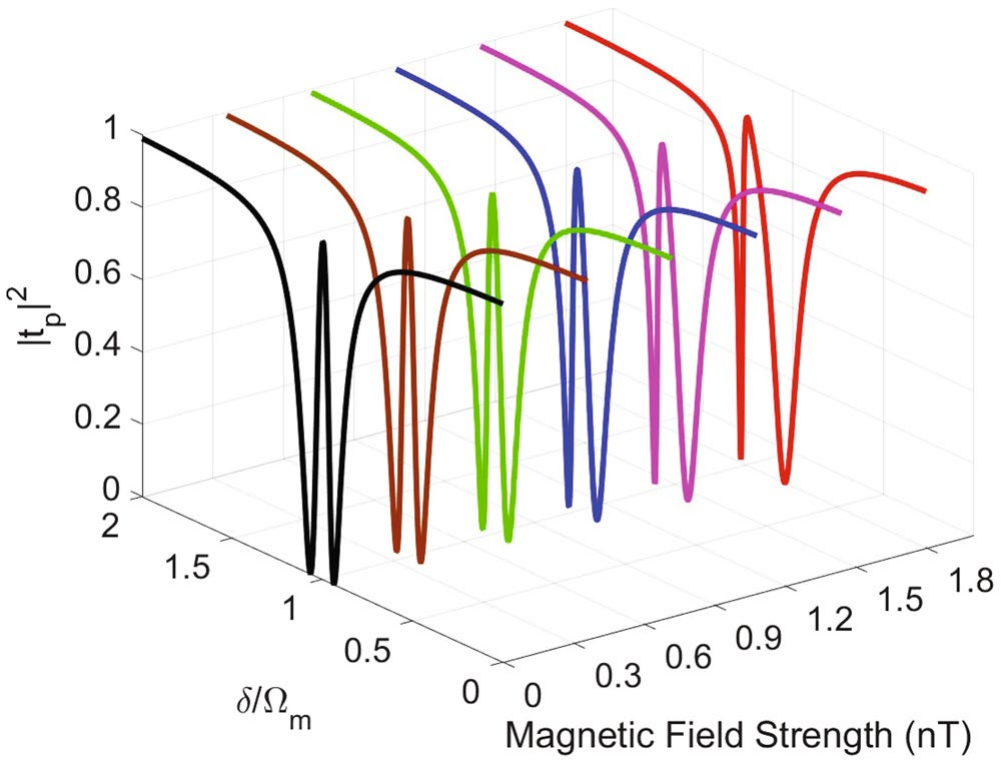
An Asymmetric transparency window in transmission spectrum of the probe field. Plots of the probe power transmission |*t*_p_|^2^ vary with *δ*/Ω_m_ and magnetic field strength *B*. The power of the control field we choose *P*_l_ = 1 *nW* and other parameters are the same as Fig. 2.

The probe power transmission |*t*_p_|^2^ as a function of *δ*/Ω_m_ and magnetic field strength *B* shows an interesting feature of OMIT induced by electromagnetic interactions in our scheme. It can be see from Fig. 3 that a general transparency window (black solid line) appears at *δ*/Ω_m_ = 1 in the absence of the electromagnetic interactions. Different from the traditional OMIT, however, the symmetry of the transparency window is broken once the electromagnetic interactions was presented and the asymmetry window is analogous to the Fano resonance window, as red solid line shown. The asymmetric transparency window becomes wider in increasing with the intensity of the magnetic field. A previous study^25^ has shown that the window widths in some special regions of the OMIT vary sharply with the charge number, which makes precise measurement of the charge number possible. In our scheme, the width of such asymmetry transparency window also shows a strong dependence on the electromagnetic interactions, which may be used to detect the value of magnetic field.

**Figure 4:**
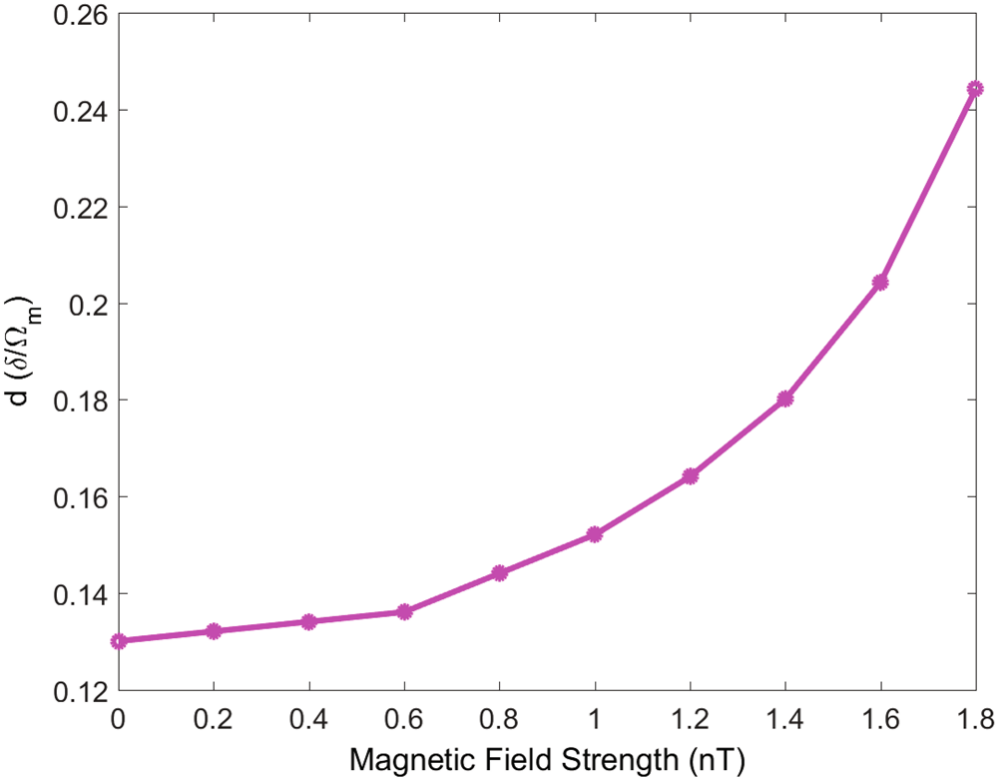
Precision measurement of the magnetic field by detecting window width *d*. Window width *d* as a function of the magnetic field strength *B*. The parameters are the same as Fig. 3.

There are two minimums in the probe power transmission shown in Fig. 3, which can be evaluated by

5 $${\frac{d{|{t}_{{\rm{p}}}|}^{2}}{d\delta }|}_{\delta ={\delta }_{-}}=0,\quad {\frac{d{|{t}_{{\rm{p}}}|}^{2}}{d\delta }|}_{\delta ={\delta }_{+}}=0,$$

where *δ*_−_ and *δ*_+_ are the two points corresponding to minimums of |*t*_p_|^2^. Here, we define the asymmetry transparency window width *d* in Fig. 3 as *d* = |*δ*_−_/Ω_m_ − *δ*_+_/Ω_m_|. In order to discuss the possibility of measuring the strength of magnetic field by the electromagnetic interactions, we plot the window width *d* as a function of magnetic field strength *B* in Fig. 4. It is shown that the window width *d* almost linearly increases with the magnetic field strength *B* within the regime $B\in [0,0.6]\,{\rm{nT}}$. However, when we consider the magnetic field strength greater than 0.6 *nT*, the growth trend of the window width *d* varying with *B* is significantly accelerated, which almost squarely increases. Such interesting relationship reminds us of the possibility of detecting the magnetic field strength *B* by measuring the separation *d* in the probe power transmission spectrum of the output field.

### Improving a measurement sensitivity of the magnetic field by adjusting the current intensity

**Figure 5:**
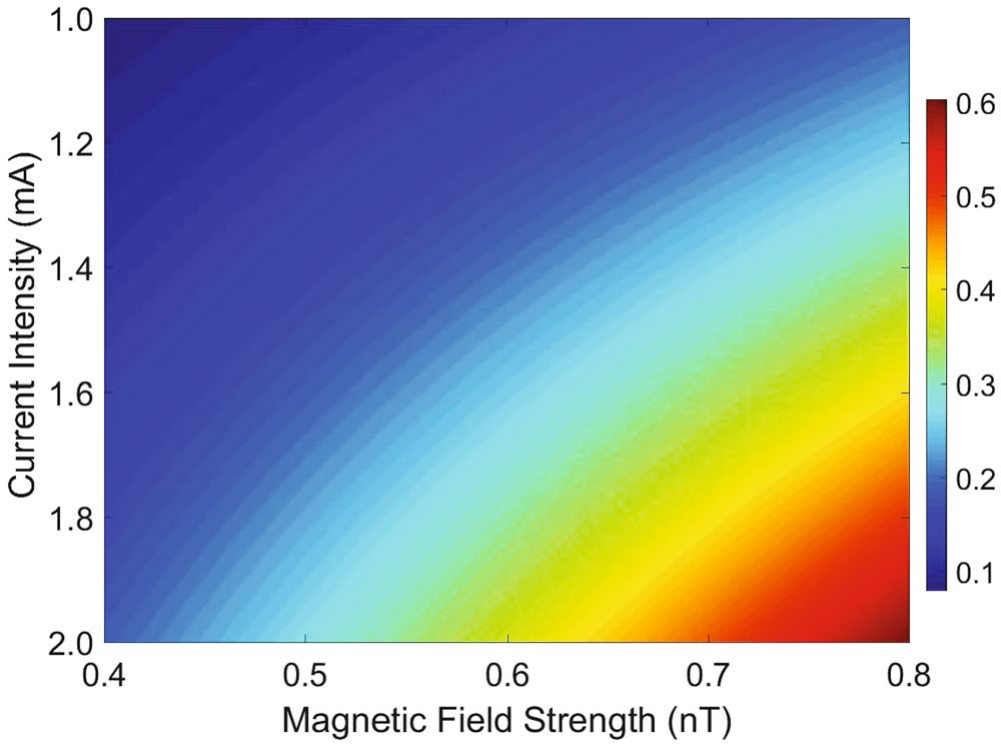
Current intensity dependent effect of the probe power transmission |*t*_p_|^2^. The probe power transmission |*t*_p_|^2^ varies with the magnetic field strength *B* and the current intensity *I*. The power of the control field we choose *P*_l_ = 10 *pW* and other parameters are the same as Fig. 2.

Up to now, we have shown that electromagnetic interactions can induce a shift of the resonance valley in the transmission spectrum of the probe field as well as an asymmetric transparency window in OMIT, which may enable a potentially practical scheme for the measurement of magnetic field. In what follows, we mainly research about how to improve the measurement sensitivity by adjusting the current intensity. The probe power transmission |*t*_p_|^2^ varying with the magnetic field strength *B* and the current intensity *I* is shown in Fig. 5. For weak electromagnetic interactions (the magnetic field strength *B* = 0.4 *nT* and the current intensity *I* = 1 *mA*), the maximal probe power transmission is quite low (about 0.1). As expected, the efficiency of the probe power transmission is enhanced when we reinforce the electromagnetic interactions in our system. The maximal |*t*_p_|^2^ increases to about 0.3 when we consider the magnetic field strength *B* = 0.6 *nT* and the current intensity *I* = 1.6 *mA*. The maximal efficiency of the probe power transmission increases as both the magnetic field strength and the current intensity increases, and the maximum values of |*t*_p_|^2^ are about 0.5 and 0.6 corresponding to the magnetic field strength *B* = 0.75, 0.8 *nT* and the current intensity *I* = 1.9, 2.0 *mA*, respectively. These results imply that resonantly enhanced feedback-backaction arising from radiation pressure can be substantively modified in the presence of electromagnetic interactions. From above discussion, the effective cavity resonance frequency ${\omega }_{{\rm{c}}}^{^{\prime} }={\omega }_{{\rm{c}}}-G\bar{x}$ depends on the applied magnetic field and the changes of the cavity field directly reflected in the probe power transmission |*t*_p_|^2^.

**Figure 6:**
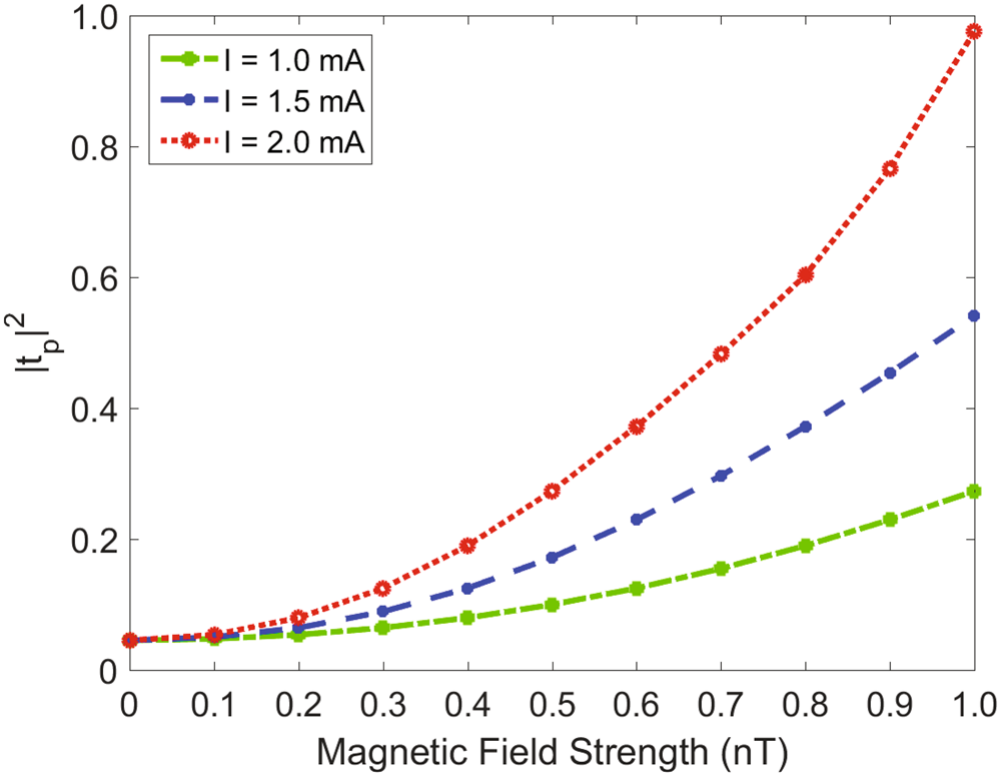
The relationship between |*t*_p_|^2^ and the magnetic field under different current intensity. Dependence of the probe power transmission |*t*_p_|^2^ on the magnetic field strength *B* with different current intensity *I*. The parameters are the same as Fig. 5.

A high dependence of the probe power transmission |*t*_p_|^2^ on the magnetic field strength *B* and current intensity *I* is shown in Fig. 6, where the relationship between |*t*_p_|^2^ and magnetic field strength *B* fits a parabolic growth pattern. Furthermore, we find that the growth trend of |*t*_p_|^2^ varying with magnetic field strength *B* is quite different for different current intensity. Such current dependent probe transmission enables an attractive device to improve the measurement accuracy in our scheme. For example, for the current intensity *I* = 1 *mA* (as the green dash-dotted line shown), the efficiency of probe power transmission |*t*_p_|^2^ changes from 0.05 to 0.27 when the magnetic field strength increasing from 0 to 1 *nT*. The growth trend of the probe power transmission varying with the magnetic field strength distinctly accelerate for a larger current (*I* = 1.5 *mA*, shown in the blue dashed line) and the maximal probe power transmission |*t*_p_|^2^ increases to about 1.0 for the current intensity *I* = 2 *mA* (shown in the red dotted line). Here, we define a sensitivity coefficient $\eta =\tfrac{{|{t}_{{\rm{p}}}|}_{{\rm{\max }}}^{2}-{|{t}_{{\rm{p}}}|}_{{\rm{\min }}}^{2}}{{|{t}_{{\rm{p}}}|}_{{\rm{\max }}}^{2}+{|{t}_{{\rm{p}}}|}_{{\rm{\min }}}^{2}}\times \mathrm{100 \% }$ which is applied to distinguish each 0.1 *nT* of the magnetic field strength. The average sensitivity coefficient *η* under different current values can be calculated, and we obtain *η*_green_ = 9.60%, *η*_blue_ = 11.26% and *η*_red_ = 14.71% corresponding to the current intensity *I* = 1.0, 1.5, 2.0 *mA*, respectively, which offer the possibility of improving the measurement sensitivity of the magnetic field strength by controlling the current values.

## Discussion

We have analyzed the transmission of the weak probe field in a hybrid optomechanical system with electromagnetic interactions. We find that the resonance valley of the probe transmission shifts obviously in the presence of the magnetic field due to modification of the effective cavity resonance frequency under the electromagnetic interactions. Furthermore, an asymmetric transparency window appears in OMIT, whose width dependents sensitively on the power of the electromagnetic interactions. Additionally, the measurement sensitivity of the magnetic field is markedly affected by current intensity. As a result, we show that our scheme provides an alternative way to measure the magnetic field with a precision of 0.1 *nT*.

## Methods

Equation (2) are intrinsically nonlinear and hard to be solved exactly. It has been shown that bistable and chaotic behavior^29^ may occur when the pump power is high enough. Similar phenomena have also been revealed in other nonlinear system with feedback, such as nonlinear optics^30^ and economic evolution^31,32^. In the present work, the pump power of about 1 nW is used, which is too low to reach the bistable region. In this case, the perturbation method can be used to solve Eq. (2) analytically, providing that the probe field is much weaker than the control field^17^. By using $a=\bar{a}+\delta a$ and $x=\bar{x}+\delta x$, where *δa* and *δx* are corresponding to the fluctuations around the steady-state solutions of the *a* and *x*, respectively. Thus, *δa* and *δx* obey the following nonlinear matrix equation:

6 $$\diamond \cdot {\rm{\Phi }}=\mu {\rm{\Phi }}+\nu {{\rm{\Phi }}}^{\ast }+\sqrt{{\eta }_{c}\kappa }{\varepsilon }_{p}{e}^{-i\delta t}\rho ,$$

where $\diamond \cdot {\rm{\Phi }}={(d\delta a/dt,\hat{{\rm{\Psi }}}\delta x)}^{T}$, Φ = (*δa*, *δx*)^*T*^, *ρ* = (1, 0)^*T*^, and

$$\mu =(\begin{array}{cc}i\bar{{\rm{\Delta }}}-\kappa \mathrm{/2} & -iG(\bar{a}+\delta a)\\ \hslash G({\bar{a}}^{\ast }+\delta {a}^{\ast }) & 0\end{array}),\,\nu =\hslash G(\begin{array}{cc}0 & 0\\ \bar{a} & 0\end{array}).$$

In this work, we mainly focus on the linear responding of our system and neglect the nonlinear terms −*iGδaδx* and −ħGδa*δa which may result in higher-order sidebands^33,34^. Next, by using the following ansatz:

7 $$\begin{array}{rcl}\,\delta a & = & {a}_{-}{e}^{-i\delta t}+{a}_{+}{e}^{i\delta t},\\ \delta {a}^{\ast } & = & {a}_{+}^{\ast }{e}^{-i\delta t}+{a}_{-}^{\ast }{e}^{i\delta t},\\ \,\delta x & = & {x}_{1}{e}^{-i\delta t}+{x}_{1}^{\ast }{e}^{i\delta t}.\end{array}$$

In the present work, we only focus on the probe field’s transmission at frequency *ω*_l_ + Ω_m_. In this sense, the matrix equations of interest are:

8 $$M\cdot \mho =N\cdot \eth+\wp .$$

where $$\mho ={({a}_{-},{a}_{+},{x}_{1})}^{T}$$, $$\eth={(\bar{a}{x}_{1},\bar{a}{x}_{1}^{\ast },\bar{a}{a}_{+}^{\ast }+{\bar{a}}^{\ast }{a}_{-})}^{T}$$, $$\eth=(-\sqrt{{\eta }_{{\rm{c}}}\kappa }{\varepsilon }_{{\rm{p}}},\,\mathrm{0,}\,0)$$ and

$$M=(\begin{array}{lll}{\rm{\Theta }}+i\delta  & 0 & 0\\ 0 & {\rm{\Theta }}-i\delta  & 0\\ 0 & 0 & \Re \end{array}),\,N=(\begin{array}{lll}iG & 0 & 0\\ 0 & iG & 0\\ 0 & 0 & -\tfrac{\hslash G}{m}\end{array}).$$

with ${\rm{\Theta }}=i{{\rm{\Delta }}}_{c}-iG\bar{x}-\kappa \mathrm{/2}$ and $\Re ={{\rm{\Omega }}}_{{\rm{m}}}^{2}-{\delta }^{2}-i{\gamma }_{{\rm{m}}}\delta$. Therefore, the solution of interest is

9 $${a}_{-}=\frac{1+if(\delta ,B)}{\kappa \mathrm{/2}-i(\bar{{\rm{\Delta }}}+\delta )+2\bar{{\rm{\Delta }}}f(\delta ,B)}\sqrt{{\eta }_{{\rm{c}}}\kappa }{\varepsilon }_{{\rm{p}}}.$$

with

$$f(\delta ,B)=\hslash {G}^{2}{|\bar{a}|}^{2}\frac{\chi (\delta )}{\kappa \mathrm{/2}+i(\bar{{\rm{\Delta }}}-\delta )},$$

and the mechanical susceptibility

$$\chi (\delta )=\frac{1}{m({{\rm{\Omega }}}_{{\rm{m}}}^{2}-{\delta }^{2}-i{\gamma }_{{\rm{m}}}\delta )}.$$

Using the input-output relation^35^, one obtains:

10 $$\begin{array}{rcl}{s}_{out}(t) & = & {s}_{in}(t)-\sqrt{{\eta }_{c}\kappa }a\\  & = & ({\varepsilon }_{l}-\sqrt{{\eta }_{c}\kappa }\bar{a}){e}^{-i{\omega }_{l}t}+({\varepsilon }_{p}-\sqrt{{\eta }_{c}\kappa }{a}_{-}){e}^{-i({\omega }_{l}+\delta )t}\\  &  & -\sqrt{{\eta }_{c}\kappa }{a}_{+}{e}^{i(\delta -{\omega }_{p})t},\end{array}$$

and the transmission of the probe beam is given by:

11 $$\begin{array}{rcl}{t}_{p} & = & \frac{({\varepsilon }_{{\rm{p}}}-\sqrt{{\eta }_{{\rm{c}}}\kappa }{a}_{-})}{{\varepsilon }_{{\rm{p}}}}\\  & = & 1-\frac{1+if(\delta ,B)}{\kappa \mathrm{/2}-i(\bar{{\rm{\Delta }}}+\delta )+2\bar{{\rm{\Delta }}}f(\delta ,B)}{\eta }_{{\rm{c}}}\kappa .\end{array}$$

which defined by the ratio of the output and input field amplitudes at the probe frequency.
